# Autoantibodies directed to centromere protein F in a patient with BRCA1 gene mutation

**DOI:** 10.1186/s13104-016-1908-7

**Published:** 2016-02-11

**Authors:** Fiona Moghaddas, Fredrick Joshua, Roberta Taylor, Marvin J. Fritzler, Ban Hock Toh

**Affiliations:** Australian Clinical Laboratories, Melbourne, Australia; Department of Rheumatology, Prince of Wales Hospital, Sydney, Australia; Department of Medicine, University of Calgary, Calgary, AB T2N 4N1 Canada; Department of Medicine, Nursing and Health Sciences, Monash University, Melbourne, Australia

**Keywords:** CENP-F, Antinuclear antibody, Malignancy, BRCA1, ALBIA

## Abstract

**Background:**

Autoantibodies directed to centromere protein F were first reported in 1993 and their association with malignancy has been well documented.

**Case:**

We present the case of a 48-year-old Caucasian female with a BRCA1 gene mutation associated with bilateral breast cancer. Antinuclear autoantibody immunofluorescence performed for workup of possible inflammatory arthropathy showed a high titre cell cycle related nuclear speckled pattern, with subsequent confirmation by addressable laser bead immunoassay of the target antigen as an immunodominant epitope at the C-terminus of centromere protein F.

**Conclusion:**

Here we review the current literature on centromere protein F, its association with breast cancer and present the first case of this antibody being identified in a person with a BRCA1 gene mutation.

## Background

Centromere protein-F (CENP-F), also known at mitosin, is a ~400 kDa nuclear protein encoded by gene 1q32–41 that associates with the centromere kinetochore complex [1, 2]. It functions in a cell cycle manner, present as a nuclear matrix protein during interphase then redistributing during early G2 of the cell cycle [1–4]. Since its discovery in 1993, a number of its functions have been elucidated, including involvement in centromere maturation, regulation of metaphase checkpoint and ensuring appropriate orientation of chromosomes [4–9]. Furthermore, it is involved in the attenuation of histone methylation and negative regulation of activating transcription factor-4 (ATF4), a transcription factor important for stress proliferation and differentiation of cells [1, 10].

The expression of CENP-F in malignancies has been investigated over the past decade, with increased immunohistochemical expression of CENP-F reported in breast, lung, ovarian and cervical cancer, non-Hodgkin lymphoma as well as squamous cell carcinoma of the oesophagus, oral mucosa and gingiva [7, 11]. Elevated expression of CENP-F has prognostic implications, correlating with clinical and pathological parameters of poor prognosis in primary breast cancer including higher standardised uptake values on positron emission tomography (PET) scan, shorter disease free survival times and higher recurrence rates [12, 13]. O’Brien et al. investigated the role of CENP-F in primary breast cancer with tissue microarrays in two cohorts and noted that CENP-F mRNA overexpression (set at >10 % based on previous studies) was significantly associated with increased tumour size, higher tumour grade and oestrogen receptor (ER) negativity [12].

Antibodies directed towards antigens expressed in tumour cells including CENP-F have been well described. Indirect immunofluorescence (IIF) of CENP-F autoantibody positive serum using Hep-2 cell line shows a nuclear speckled pattern with cell cycle dependent fluorescence and negative chromatin mass in mitosis [14]. Although present in less than 1 % of patients with malignancy, anti-CENP-F antibodies have a positive predictive value of approximately 50 % for cancer [14–16]. Furthermore, there is evidence to suggest that the appearance of these antibodies may be seen during progression from benign to malignant processes, as was demonstrated in two patients peri transition from chronic hepatitis to hepatocellular carcinoma as well as in oral leukoplakia, a premalignant lesion of the oral mucosa [17–19].

Previously, anti-CENP-F antibodies have been detected using radioimmunoassay, immunoblotting with native recombinant antigens or enzyme linked immunosorbent assay [16]. A more recent development has employed an addressable laser bead immunoassay (ALBIA) based on the two principal immunogenic epitopes of CENP-F, peptides F1 and F4 [14, 20, 21]. Interestingly, half of patients who tested positive for CENP-F using this method did not have the corresponding typical IIF pattern, suggesting that IIF may be an insensitive screening method for this autoantibody, and that the incidence in patients with malignancy may indeed be more than 1 %.

The potential therapeutic benefit of detecting increased expression of CENP-F, or autoantibodies directed towards it, has been studied with the use medications such as zoledronic acid that target farnesyl diphosphate synthase [22]. CENP-F is a farnesylated protein and inhibition of farnesylation leads to loss of CENP-F from the kinetochore and subsequent inactivation [22]. Farnesyl transferase inhibitors (FTIs) are a novel class of chemotherapeutic agents showing promise in the treatment of cancer [23]. Although thought to act mainly by its downstream effects on Ras, the actions of FTIs are not Ras-specific and it is possible that the lack of farnesylation of other proteins, including CENP-F, may have a role in their success [12, 23–26].

To date, neither the over expression of CENP-F nor the detection of autoantibodies directed towards it have been described in a BRCA1 mutation positive population. BRCA1, a tumour suppressor gene located on chromosome 17, is mutated at high frequency in hereditary breast and ovarian cancers. The role of CENP-F in pathogenesis of disease in this population, and the theoretical benefit of FTIs, has not yet been explored.

## Case report

A 48-year-old Caucasian female was referred to a rheumatologist experiencing fatigue and widespread joint pain, worse in the morning and improving with activity, associated with new onset psoriasis. The results from a number of investigations performed 3 months prior to review include C-reactive protein 16 (n < 5 mg/L), erythrocyte sedimentation rate 57 (n < 20 mm/h), and a negative rheumatoid factor and HLA-B27. An antinuclear antibody (ANA) performed at this time was reported as positive at a titre of 1:1280 with a speckled pattern, but antibodies to extractable nuclear antigens (ENA) and ds-DNA were negative.

**Fig. 1:**
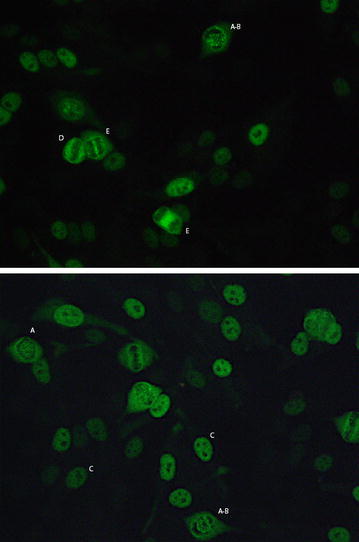
Cell cycle dependent pattern characterised by strong centromere staining in prophase (***A***) and prometaphase (***A***, ***B***), variable nuclear staining in interphase (***C***), weak staining in metaphase (***D***) and staining of midbody during anaphase and early telophase (***E***). Hep-2000 Fluorecent ANA-Ro Test System (Immuno Concepts), 1:160, 40× magnification

The ANA was repeated by IIF on the HEp-2000 Fluorescent ANA-Ro60 Test System according to the manufacturer’s directions (Immuno Concepts, Sacramento, CA). The sample was screened at a dilution of 1:160 and sequentially diluted at titres 1:320, 1:640 and 1:1280 with the result recorded as the highest dilution ANA pattern remained discernible. The repeat ANA showed a nuclear speckled-II (NSII) pattern [27] at a titre of 1:1280 (Fig. 1). The NSpII pattern is a cell cycle dependent pattern suggestive of CENP-F autoantibodies, typically characterised by strong centromere staining in prometaphase and metaphase, variable nuclear staining in interphase and weak staining in anaphase. Approximately 60 % of sera demonstrating this pattern also have staining of the midbody during telophase (Fritzler unpublished data). As there were no commercially available assays identifying this specificity, the sample was sent to the Mitogen Advanced Diagnostics Laboratory at the University of Calgary where an ALBIA was developed for this purpose using protocols as previously described [28]. This process involved covalent coupling CENP-F peptides F1 and F4 to separate dye laden addressable microbeads and pipetting both into a single microtiter well, with the subsequent addition of both serum and fluorochrome-coupled secondary antibody. The sample was then processed by a Luminex 200 analyser (Luminex Corp., Austin, TX). The two lasers in the instrument and flow cytometric principles allowed determination of both specificity and quantification of autoantibody, with results expressed as mean fluorescence units (MFU) of the fluorochrome-coupled secondary antibody [28]. Antibodies to both epitopes were detected in the patient’s serum, with a low positive reading for p-F1 and a high positive for p-F4.

Given the association of this autoantibody specificity with malignancy, further history was sought from the referring clinician. At age 45, the patient was diagnosed with stage III left sided breast cancer and underwent mastectomy, axillary clearance and combined adjuvant chemotherapy and radiotherapy. Her post-treatment course was complicated by melanoma in the field of radiation. She underwent genetic testing for risk stratification and was noted to be BRCA1 positive. Approximately 1 year after the initial diagnosis she underwent a prophylactic bilateral salpingo-oophorectomy, with two foci of severe dysplasia seen in the right fallopian tube. Six months later, she was noted to have lymphadenopathy in the contralateral axillae that was biopsied and positive for adenocarcinoma, and subsequently had a right mastectomy and axillary clearance.

## Discussion

There have been no reports of antibodies to CENP-F expressed in patients who are BRCA1 gene mutation positive. Literature on the association of CENP-F immunohistochemical staining in breast cancer of patients with BRCA1 mutation is also lacking. There are a number of potential reasons for this, including the questionable value of detecting this marker of high risk in a population that will already undergo rigorous monitoring for recurrence post diagnosis. Furthermore, the genetic heterogeneity in breast cancer may lead to the presumption that the co-existence of these autoantibodies may indeed be coincidental, and antibodies to multiple autoantigens may be detected if tested. In an unselected breast cancer population, Fritzler et al. documented 7/190 (3.7 %) of breast cancer patients testing positive for CENP-F autoantibodies [14]. The rate of positivity in the BRCA1 population is unclear, and cohort studies may help clarify this. The implication of this information, however, is less certain.

There is little information on the use of these autoantibodies as a marker of disease remission or recurrence. One report identified a single patient with breast cancer and undifferentiated connective tissue disease and tracked the mean fluorescence units (MFU) of autoantibodies targeting both p-F1 and p-F4 over time [14]. The relationship between autoantibody levels, epitope specificity and the clinical picture was not clear. As these autoantibodies are unlikely to be singularly causal, we cannot necessarily expect to find such a linear association. Whether there is utility in monitoring antibodies to CENP-F in patients who have tested positive remains to be determined. There have been no reports of patients having CENP-F positivity reverting to negative, and there is insufficient data on the semi quantitative and quantitative measures in individual patients over time. It is difficult to determine the future risk of malignancy in our patient, and although the positive predictive value of malignancy in those testing positive is 50 %, there is no literature on whether this risk changes after detection and treatment of an initial malignancy.

As mentioned above, there are theoretical benefits of FTI in patients with malignancies who express autoantibodies to CENP-F. This is difficult to study directly due to small numbers of patients with CENP-F antibodies and FTIs currently being in trial, but it does raise the prospect of this class of agent being effective in this population. Our patient has already been exposed to multiple chemotherapeutic agents and it is foreseeable that she may require treatment for further malignancies. This may prove to be a useful option in the future.

## Conclusion

This case highlights the association of CENP-F with malignancy, as well as deficiencies in our current knowledge of autoantibodies targeting this antigen. The incidence of these autoantibodies in patients with a BRCA1 mutation is unknown. Further research into the prevalence in the BRCA1 population may provide useful information for patient care as the target antigen is implicated in the mechanism of action of chemotherapeutic agents.

## Consent

Written informed consent was obtained from the patient for publication of this Case report and any accompanying images. A copy of the written consent is available for review by the Editor of this journal.

## Abbreviations

ALBIA: addressable laser bead immunoassay
ANA: antinuclear antibody
ATF4: activating transcription factor-4
BRCA1: breast cancer 1
CENP-F: centromere protein F
ENA: extractable nuclear antigens
ER: oestrogen receptor
FTI: farnesyltransferase inhibitors
IIF: indirect immunofluorescence
MFU: mean fluorescence units
NSII: nuclear speckled-II pattern
PET: positron emission tomography
